# Apparent Digestibility Coefficients of Selected Plant Protein Feed Ingredients and Feeds for Coho Salmon (*Oncorhynchus kisutch*) Postsmolts Cultured in Fresh Water

**DOI:** 10.1155/anu/3047597

**Published:** 2025-07-28

**Authors:** Baobin Lu, Leyong Yu, Hairui Yu, Abdur Rahman, Chengyu Ma, Xiaojing Wu, Lingyao Li, Shahid Sherzada, Nimra Hussain

**Affiliations:** ^1^College of Fisheries and Life Science, Shanghai Ocean University, Shanghai 201306, China; ^2^Key Laboratory of Biochemistry and Molecular Biology in Universities of Shandong (Weifang University), Weifang Key Laboratory of Coho Salmon Culturing Facility Engineering, Institute of Modern Facility Fisheries, College of Biology and Oceanography, Weifang University, Weifang 261061, China; ^3^Shandong Freshwater Fisheries Research Institute, Jinan 250013, China; ^4^College of Fisheries, Huazhong Agricultural University, Wuhan 430070, China; ^5^Weifang Key Laboratory of Salmon and Trout Health Culture, Conqueren Leading Fresh Science and Technology Inc. Ltd., Weifang 261205, China; ^6^Department of Animal Sciences, University of Veterinary and Animal Sciences, Jhang Campus, Lahore 54000, Pakistan; ^7^Shandong Collaborative Innovation Center of Coho Salmon Health Culture Engineering Technology, Shandong Conqueren Marine Technology Co. Ltd., Weifang 261108, China; ^8^Department of Zoology, Government College University, Lahore 54000, Pakistan; ^9^Department of Fisheries and Aquaculture, University of Veterinary and Animal Sciences, Lahore 54000, Pakistan

**Keywords:** amino acid digestibility, apparent digestibility coefficients (ADCs), Coho salmon, feed efficiency, proximate composition

## Abstract

The aquafeed industry relies on fish meal as a major protein source, but its use raises economic and environmental concerns, prompting the search for sustainable alternatives. This study compared the apparent digestibility coefficients (ADCs) of dry matter (DM), energy, protein, phosphorus, lipids, and amino acids (AAs) for selected protein ingredients in Coho salmon. For this purpose, one reference and seven test diets corresponding to beer yeast (BY), corn gluten meal (CGM), cottonseed meal (CSM), peanut meal (PNM), rapeseed meal (RSM), soybean meal(SBM), and soy protein concentrate (SPC) were formulated with the ratio of 70:30 of the reference diet and one of the test ingredients. 1200 fish were randomly distributed into 24 glass aquaria (each with a diameter of 3.0 m and a depth of 1.5 m, water volume 8.5 m³) with three aquaria per experimental diet (total *n* = 50*⁣*^*∗*^24 = 1200). The experiment lasted for 8 weeks. The results indicated that ADCs of DM ranged from 43.34% for RSM to 72.90% for SPC. Similarly, the highest ADCs of crude protein (72.69% to 86.76%), lipid (77.09% to 87.72%), and gross energy (74.12% to 55.44%) were observed in SPC, and the lowest were found in RSM. However, the ADCs of phosphorus ranged from 36.14% (RSM) to 47.35% (SBM). The ADCs of proximate nutrients for BY, CGM, PNM, SBM, and SPC were significantly higher (*p*  < 0.05) than other protein ingredients (RSM, CGM). Similar patterns of digestibility were observed for individual AAs of the test ingredients. Overall, the diet containing SPC appeared to be more suitable and compatible with the reference diet. Conversely, PNM, BY, SBM, and CGM showed some potential as aquafeed ingredients, whereas CSM and RSM appear to be the least viable options. Such information aids in better feed formulation by focusing on nutrient absorption rather than raw ingredient composition.

## 1. Introduction

Aquaculture is the fastest-growing food sector, driven by intensive farming utilizing formulated feeds. Aquaculture production of aquatic animals reached 94.4 million tons, accounting for 51% of the total aquatic animal production, surpassing capture fisheries for the first time. This milestone underscores the significant role aquaculture now plays in global seafood supply [1]. Salmon farming is valued at $15.6 billion globally, expanding at a rate of 7% annually [2]. This fast expansion may bring sustainability challenges related to feed resources, as their sourcing and composition affect both environmental and economic viability [3]. Coho salmon (*Oncorhynchus kisutch*) is a highly valued temperate fish native to the North Pacific Ocean [4]. Their nutritional requirements are well established; however, their conventional dependency on fish meal and fish oil as a main protein source in salmonid feed [5] may impart economic and environmental concern.

Fishmeal is a key protein source for carnivores due to its balanced amino acids (AAs), high phosphorus, digestibility, palatability, and lack of antinutritional factors (ANFs) [6, 7]. While fish meal remains a significant protein source in aquaculture due to its nutritional profile, its proportion in modern salmonid diets has indeed declined substantially in recent years. Current studies revealed that plant-based proteins (e.g., soybean and canola) and novel ingredients (e.g., insect meal, and microbial proteins) now dominate salmonid feed formulations, with fish meal inclusion rates often reduced to <10%–15% in commercial diets. This shift reflects efforts to improve sustainability and reduce reliance on marine resources [8].

Aquaculture nutritionists are continuously in search of alternative protein sources to reduce reliance on fishmeal. Although no single ingredient can fully replace it. However, diverse options from animal, plant, and microbial origins are in consideration. These alternatives must support optimum fish growth, feed efficiency, and health along with long-term sustainability [9].

Plant-based protein sources, such as soybean, corn, cottonseed, peanut, and so on came out to be promising substitutes for fishmeal in the aquafeed industry, offering potential benefits. These are attractive due to their consistent nutrient content, availability, and affordability, but their use is limited due to the presence of certain ANFs, lack of some AAs, and low digestibility due to starch and fiber contents [6, 10]. Moreover, these ANFs also pose significant challenges for the digestion and absorption of nutrients [3]. Besides this, key factors, that is, amino acid profile, energy density, fiber, and nutrient absorption should be kept in mind, while replacing fishmeal with plant proteins [6].

The actual knowledge about the digestibility of these ingredients is imperative for optimization of aquafeeds. The optimization of apparent digestibility coefficients (ADCs) plays an important role in enhancing feed efficiency, reducing environmental impact, and promoting sustainable aquaculture [11]. Furthermore, it enables better feed formulation by focusing on nutrient absorption rather than raw ingredient composition [12]. Taking into account the importance of digestibility for feed formulation, this study was designed to compare the apparent nutrient digestibility of proximate nutrients and amino acid profiles of different protein sources for postsmolt Coho salmon (*Oncorhynchus kisutch*) cultured in freshwater.

## 2. Materials and Methods

### 2.1. Ingredients Preparation and Diet Formulation

The plant feed ingredients tested for ADCs included beer yeast (BY), corn gluten meal (CGM), cottonseed meal (CSM), peanut meal (PNM), rapeseed meal (RSM), soybean meal (SBM), and soy protein concentrate (SPC) and were obtained from Shandong Conqueren Marine Technology Co., Ltd., Weifang 261108, China. Seven test diets were prepared by mixing a reference diet (Table 1) and one of the test ingredients at a ratio of 70:30 according to the method of Cho and Slinger [13]. The reference diet was formulated to get 450 g/kg crude protein and 175 g/kg crude lipid. Yttrium oxide (Y_2_O₃, 0.5 g/kg) was incorporated into test and reference diets as an inert marker. The proximate and amino acid compositions of the test ingredients and diets are shown in Tables 2 and 3, respectively.

Each of the test ingredients was ground into fine powder through 150 mm mesh with the help of a vertical ultrafine grinder (SWFL 130E, Jiangsu Muyang Group Co., Ltd., Liyang, China). All diets were prepared as cold extrusion pellets containing approximately 30% moisture. Pellet size was 3.0 mm. After extrusion, the pellets were air-dried at 25°C for approximately 48 h before storage at −20°C.

### 2.2. Animals and Experimental Procedures

Coho salmon postsmolts (*n* = 1200) in this trial were obtained from one base of the Shandong Collaborative Innovation Center of Coho Salmon Health Culture Engineering Technology, Linyi, China. Prior to the start of the experiment, the postsmolts were reared in an indoor concrete pond (12.0 m length × 6.0 m width × 2.5 m height) and fed with commercial salmon feed (Shandong Conqueren Marine Technology Co., Ltd., Weifang, China) containing 450 g/kg of crude protein and 200 g/kg of crude lipid, with a 3.0 mm diameter. Feeding was done three times daily (7:00, 12:00, and 17:00) for 2 weeks. This was followed by feeding the reference diet to acclimate the fish to experimental conditions. Afterward, the fish were fasted for 24 h, and 50 fish of each weighing 183.17 ± 3.29 g were randomly distributed into 24 glass aquaria (each with a diameter of 3.0 m and a depth of 1.5 m, water volume 8.5 m³) with three aquaria per experimental diet (total *n* = 50*⁣*^*∗*^24 = 1200). During the experimental period of 8 weeks, the fish were manually fed to apparent satiation three times (7:30, 13:00, 17:30) a day. Water temperature ranged from 15.0 to 16.0°C, pH ranged from 7.1 to 7.5, and dissolved oxygen was above 7.5 mg/L. The postsmolts were reared under a natural light regime.

### 2.3. Feces Collection

Fecal samples were collected weekly during weeks 3–6 of the 8-week trial, with each fish stripped only once per week (maximum four times total). Fish were fasted for 24 h prior to each stripping event and allowed a 48-h recovery period under optimal conditions (DO >7.5 mg/L, 15–16°C). This protocol, approved by the IACUC (No. 20210413007), aligns with established salmonid digestibility studies and ensured fish welfare, as evidenced by no observed mortality or behavioral abnormalities. Collected samples from each aquarium were pooled and stored at −20°C until analysis.

### 2.4. Analytical Methods

The proximate composition of fish diets and their feces is ground for homogenization. Methods given by the Association of Official Analytical Chemists (AOAC) [14] were used for proximate analysis. First, dry matter (DM) contents were determined by drying the samples at 105°C. In addition to that, the Kjeldahl method was used for measuring nitrogen (N × 6.25) for the determination of crude protein contents, while crude lipids were measured by ether extraction using the Soxhlet method. On the other hand, ash contents were calculated by heating the samples at 550°C for 24 h in a muffle furnace. After perchloric acid digestion, an inductively coupled plasma–atomic emission spectrophotometer (ICP-OES, VISTA-MPX, VARIAN, USA) was used to analyze the yttrium and phosphorus in the feed and feces samples. The gross energy content was determined using an automatic Parr 1281 oxygen bomb calorimeter (Parr, Moline, IL, USA). The AAs were analyzed using an automatic analyzer (Model A300, MembraPure GmbH, Frankfurt, Germany).

### 2.5. Calculations and Statistical Analyses

For data analysis and interpretation, following calculations were carried out:

ADCs of experimental diets were determined by following Maynard and Loosli [15]:

  $$\begin{array}{c} \text{ADC of dry matter }\left(\text{\%}\right)\;=\;\left(100-\left(\text{\% yttrium in feed}/\text{\% yttrium in feces}\right)\times 100\right). \end{array}$$

  $$\begin{array}{c} \text{ADC of nutrient}/\text{ energy }\left(\text{\%}\right)\;=\;\left(100-\left(\text{\% yttrium in feed }/\text{\% yttrium in feces}\right)\;\times \;\left(\text{\% nutrient or energy in feces }/\text{\% nutrient or energy in feed}\right)\;\times 100\right). \end{array}$$

ADCs of test ingredient were determined by given formula as follows [16]:

  $$\begin{array}{c} \text{ADC of test ingredient }\left(\text{\%}\right)\;=\;100/30\times \left(\text{ADC in test diet }-\;0.7\text{ ADC in reference diet}\right). \end{array}$$

All data are expressed as the means ±standard deviation (SD) and analyzed using SPSS version 25.0 software (SPSS Inc., Chicago, IL, USA). Statistically significant observations were performed using one way ANOVA followed by Tukey's test, which was used to compare the means among individual treatments, the significant difference was set as *p*  < 0.05.

## 3. Results

### 3.1. Comparative Analysis of Reference and Test Diets

Table 4 demonstrated that the highest DM digestibility was found in diets containing SPC (69.07%) and BY (68.26%), while the lowest was in diets having RSM (60.20%) and CSM (61.06%). On the other hand, crude protein and lipid contents were significantly (*p*  > 0.05) lower in diets containing CSM and RSM as compared to other tested diets. Regarding phosphorus contents, the salmon group that was fed with the reference diet showed the highest digestibility (72.05%), while RSM (61.28%) and CSM (61.62%) showed the lowest. Similarly, the gross energy ADC value was also found highest with the reference diet (79.29%), followed by SPC (77.74%) and SBM (77.50%), while RSM (72.14%) and CSM (72.79%) were the lowest. Additionally, amino acid digestibility values for different diets were found similar to those of crude protein contents. Diets containing RSM and CGM showed significantly (*p*  > 0.05)lower amino acid ADC values as compared to other diets.

### 3.2. Apparent Digestibility of Proximate Nutrients

ADCs of proximate nutrient, phosphorus, and gross energy of the test ingredients vary significantly across different feed ingredients (Table 5). The digestibility of DM followed the pattern RSM < CSM < SBM < PNM < CGM < BY < SPC with the lowest value (43.34%) for RSM and highest (72.90%) for SPC. Likewise, highest protein, lipid, and gross energy ADCs values for SPC were significantly greater than the other tested ingredients, indicating that SPC as a good quality feed ingredient that had highly nutrients utilization for Coho salmon as compared to other studied feed sources. ADCs of protein for the test ingredients were ranging from 72.69% to 86.76%, with the highest value observed in SPC and lowest in RSM just like ADCs of DM. Similarly, ADCs of crude lipid and gross energy ranged from 77.09% to 87.72% and 74.12% to 55.44% respectively, with the highest value observed in SPC, while lowest in RSM. ADCs of protein in SBM, CGM, BY, PNM, and SPC were significantly higher than other feedstuffs. However, the ADCs of phosphorus ranged from 36.14% to 47.35%, with the lowest value reported for RSM and highest for SBM.

Moreover, it illustrated the amino acid ADCs values for different AAs present in test ingredients. It indicated that their digestibility values followed the similar pattern as that observed for crude protein. ADC values for arginine (Arg), histidine (His), leucine (Leu), lysine (Lys), phenylalanine (Phe), tyrosine (Tyr), valine (Val), aspartic (Asp), glutamic (Glu), serine (Ser) and proline (Pro) in SPC were higher than those of other feed ingredients. Moreover, among all test ingredients, the ADCs values of in RSM and CSM were lower than other ingredients.

## 4. Discussion

This study explored the ADC values of commonly used feed ingredients, that is, BY (BY), CGM, PNM, SBM, RSM, CSM, and SPC as potential fishmeal replacements for postsmolts Coho salmon (*Oncorhynchus kisutch*) cultured in freshwater, providing valuable insights about feed formulation and sustainability of aquafeeds.

### 4.1. Apparent Digestibility of Proximate Nutrients

The digestibility coefficients of studied test ingredients undergo significant variation, with the smaller values being associated with those having low protein contents, indicating that those ingredients that were high in protein contents were easily digestible by salmon. It confirmed that high carbohydrate and fiber content in plant protein sources provides a negative impact on DM digestibility [17]. Moreover, low digestibility could be due to high carbohydrates, due to which salmon struggle to break them down. This is attributed to rapid transit of indigestible carbohydrates through the digestive system along with intact proteins, which affect their digestion efficiency [18].

Moreover, the cold extrusion processing method in our study likely limited starch gelatinization and fiber degradation, contributing to the moderate DM (43.34%–72.90%) and energy (55.44%–74.12%) ADCs observed. This aligns with recent findings that cold-extruded plant-based diets for salmonids exhibit lower starch digestibility due to retained starch crystallinity [19], while thermal processing (≥100°C) significantly improves carbohydrate utilization by breaking down resistant starch and fiber [20]. The high carbohydrate content in ingredients like CGM (304.6 g/kg) and CSM (469.3 g/kg) may have further reduced nutrient absorption by accelerating gut transit, as demonstrated in rainbow trout fed cold-extruded diets [21]. These processing limitations highlight the need to explore thermal alternatives or carbohydrase supplementation [22] to optimize Coho salmon feeds, particularly for starch-rich ingredients.

In this study, DM digestibility of SPC, CGM, and PNM is significantly higher than other feed ingredients, i.e., CSM and RSM. Likewise, lower digestibility of DM for RSM and CSM as compared to PNM, SBM, and CGM (30% of the test ingredient) was noticed by Mo et al. [23] in mandarin fish, *Siniperca chuatsi*. Similar results of DM digestibility for SBM were also recorded by Asad et al. [24] in *Labeo rohita*. Previously, Egerton et al. [25] observed 70.77% of the ADC value of DM as they fed Atlantic salmon (*Salmo salar*) with a diet having 80% plant protein supplemented with 15% fishmeal.

Regarding crude protein, all ingredients showed efficient protein digestibility, that is, having ADC values above 80%, except for RSM and CSM (72.69 and 73.32, respectively), probably due to low protein content in them. Furthermore, the ADC values for DM and crude protein of RSM for juvenile *Pseudobagrus ussuriensis* were greater than the values documented for Coho salmon in this study, implying that *Pseudobagrus ussuriensis* can efficiently digest RSM [26].

Our findings match well with what other researchers have found in salmonids. For RSM, we saw low phosphorus digestibility (36.14%), similar to the 30%–40% range reported in earlier studies [27]. It happens due to the fact that RSM contains phytate that blocks phosphorus absorption. SBM showed better protein utilization (85.57% ADC), consistent with findings that proper thermal processing reduces trypsin inhibitors [28]. The superior performance of SPC (86.76% protein ADC) further confirms that additional processing mitigates ANFs [29]. However, our CSM results (73.32% protein digestibility) were lower than expected, probably because of remaining gossypol (a natural toxin in cottonseed) that other salmon studies [30] have also found problematic. These comparisons show that while some plant proteins can work well in salmon feeds, others like RSM and CSM need special treatment to make them more digestible. Madrid et al. [31] evaluated the digestibility of different plant and animal-based ingredients in diets of *Totoaba macdonaldi* and found much lower DM and protein ADCs values for SBM, SPC, and CGM as compared to the current study. Such differences are characterized by specie specific nature to digest certain kind of feed ingredients. Furthermore, the protein ADC value of 87% obtained for SPC in the current study was slightly lower than values typically reported for salmonid species (>92%). This incongruity may be partially attributed to our use of manual stripping for fecal collection, a method that has been shown to potentially underestimate true digestibility coefficients compared to other collection techniques [16, 32]. Besides methodological limitations, manual stripping remains a widely used technique in digestibility studies due to its practicality and direct comparability with numerous previous studies employing similar methods [33, 32].

ADCs of crude lipid of all test ingredients ranged from 77.09 to 87.72, signifying the better digestion of lipid contents. The highest digestibility of lipid was seen in SPC, while its proximate composition showed lower lipid levels of 16.8 g/kg. On the other hand, test ingredient CGM presented about double the amount of lipid content (32.1 g/kg) as that present in SPC, but its ADC value was less than that of SPC. It means salmon faces difficulty in digesting the lipid content of CGM, while it has better digestion efficiency for SPC. According to Chi et al. [34], the digestibility of crude lipids depends on fatty acid chain length, unsaturation level, and other feed components. Additionally, there occurs another interesting fact regarding BY, as it shares almost equal lipid ADC value with SPC, with only 5.3% crude lipid content. It further demonstrated the complexity of the digestion process, as it is not only dependent upon the lipid profile but also influenced by other components present in feed.

The ADC value of gross energy for RSM, CSM, and CGM was lower than that of other test ingredients, probably due to the presence of high ash content in these ingredients. In comparison, the gross energy ADC value for RSM is inconsistent with the study of Chu et al. [35], who determined ADC values of different protein ingredients, including RSM, in loach (*Misgurnus anguillicaudatus*). Additionally, Yu et al. [36] determined that the DM, crude protein, crude lipid, phosphorous, and energy digestibility values of CSM for juvenile snakehead (*Ophiocephalus argus*) were higher as compared to this study, which is attributed to the better digestion capability of snakehead for CSM. Phosphorus is a key pollutant in freshwater; therefore, improving phosphorus digestibility and reducing its excretion can help in lowering the environmental pollution [26]. The current study observed that Coho salmon postsmolts may be more efficient at digesting phosphorus from BY, CGM, PNM, SBM, and SPC but may hardly utilize phosphorus from CSM and RSM. These results were highly reflective of the finding of Che et al. [26], who studied ADCs of various feed ingredients for juveniles of *Pseudobagrus ussuriensis*.

### 4.2. Apparent Digestibility of AAs

The amino acid ADCs for all test ingredients were above 80% except for RSM and CSM, which showed significantly lower ADC values than other ingredients. Likewise, lower ADC values for AAs of RSM and CSM were documented by Che et al. [26] in juveniles of *Pseudobagrus ussuriensis*. For comparison, Burr et al. [37] also described higher ADC values (above 90%) for CGM, SPC, and SBM when fed to Atlantic salmon, *Salmo salar*, at a rate of 30% of the diet by weight. ADC values of individual AAs within the feed ingredient undergo significant variations among different test ingredients. Methionine, an essential amino acid, showed the lowest digestibility in RSM, regardless of enough concentration in the amino acid profile. A similar trend of methionine digestibility among RSM, PNM, and SBM was observed by Mo et al. [23] in mandarin fish, *Siniperca chuatsi*. However, Yu et al. [36] determined a much greater ADC value of methionine (82%) in juvenile snakehead (*Ophiocephalus argus*), representing improved digestion, probably due to species-specific nature.

### 4.3. Comparative Analysis of Reference and Test Diets

The test diets comprising plant-based ingredients exhibited flexible digestibility coefficients (ADCs) when compared to the fishmeal-based reference diet. SPC was found to be the most compatible alternative, demonstrating high protein (86.76%) and lipid (87.72%) ADCs, comparable to values described for King salmon (*Oncorhynchus tshawytscha* ≥90%) [20]. This aligns with findings in olive flounder, where SPC enhanced growth and nutrient consumption [38]. The superior performance of SPC in feed is attributed to its low ANF content and balanced amino acid profile.

In contrast, RSM and CSM displayed the lowest digestibility, particularly for phosphorus (36.14%–37.29%) and DM (43.34%–46.19%). Similar limitations were detected in European seabass, where RSM's glucosinolates reduced nutrient utilization [39], and in Asian seabass, where CSM's gossypol compromised digestibility [40]. These results claim the need for ANF mitigation strategies, such as fermentation or enzymatic treatment, to boost their utility in aquafeeds.

BY and CGM portrayed intermediate performance. BY showed unexpectedly high lipid ADC (85.23%) despite its low crude lipid content (5.3 g/kg), suggesting synergistic effects with other dietary components. CGM possessed lower lipid digestibility (81.27%) compared to SPC, despite its higher lipid content. This reflects species-specific metabolic differences, as observed in cobia fish [41].

## 5. Conclusion

This study portrayed information about the digestibility of proximate nutrients and AAs in tested feed ingredients for the culturing of Coho salmon (*Oncorhynchus kisutch*) postsmolts. It inferred that feed containing BY, CGM, PNM, SBM, and SPC exhibits higher nutrient digestibility as compared to CSM and RSM. Moreover, the diet containing SPC appeared to be more suitable and compatible with the reference diet. Conversely, PNM, BY, SBM, and CGM showed some potential as aquafeed ingredients, whereas CSM and RSM appear to be the least viable options. However, the effects of these ingredients on the growth performance of Coho salmon require further investigation. Such information aids in better feed formulation by focusing on nutrient absorption rather than raw ingredient composition. Furthermore, it provides fundamental knowledge for future studies on ingredient replacements and nutrient needs of Coho salmon (*Oncorhynchus kisutch*) postsmolts, necessary for optimizing performance and minimizing the costs and waste.

## Figures and Tables

**Table 1:** Reference diet formulation for determination of apparent digestibility coefficients in Coho salmon (*Oncorhynchus kisutch*) postsmolts.

Ingredient	Concentration (g/kg DM)
Peruvian fish meal^a^	500.0
Poultry by-product meal^a^	100.0
Antarctic krill meal^a^	80.0
High gluten wheat flour^a^	140.5
α-starch^a^	25.0
Fish oil^a^	80.0
Soybean oil^a^	50.0
Monocalcium phosphate^a^	10.0
Vitamin premix^b^	5.0
Mineral premix^c^	5.0
Choline chloride^a^	3.0
Ascorbic acid phosphate^a^ (350 g/kg)	1.0
Yttrium oxide (Y_2_O_3_)	0.5

^a^Provided by Shandong Conqueren Marine Technology Co., Ltd., Weifang, China.

^b^Composition (IU or g/kg vitamin premix): retinal palmitate, 10,000 IU; cholecalciferol, 4000 IU; α-tocopherol, 75.0 IU; menadione, 22.0 g; thiamin-HCl, 40.0 g; riboflavin, 30.0 g; D-calcium pantothenate, 150.0 g; pyridoxine-HCl, 20.0 g; meso-inositol, 500.0 g; D-biotin, 1.0 g; folic acid, 15.0 g; niacin, 300.0 g; cyanocobalamin, 0.3 g.

^c^Composition (g/kg mineral premix): AlK(SO_4_)_2_•12H_2_O, 123.7; CuSO_4_•5H_2_O, 32.0; CoCl_2_•6H_2_O, 49.0; FeSO_4_•7H_2_O, 707.0; MgSO_4_•7H_2_O, 4317.0; MnSO_4_•4H_2_O, 31.0; KI, 5.3; NaCl, 4934.0; Na_2_SeO_3_•H_2_O, 3.4; ZnSO_4_•7H_2_O, 177.0.

**Table 2:** Proximate and amino acid composition (g/kg) of the test ingredients.

Test Ingredients	BY^a^	CGM^a^	CSM^a^	PNM^a^	RSM^a^	SBM^a^	SPC^a^
Proximate analysis (g/kg dry matter)							
Dry matter	947.4	924.5	888.8	912.5	888.4	878.3	930.9
Crude protein	505.2	647.8	440.7	510.8	415.3	497.2	706.1
Crude lipid	5.3	32.1	21.3	24.8	16.3	22.2	16.8
Ash	45.4	15.5	68.7	65.5	69.9	64.1	38.8
Phosphorus	10.0	7.7	11.1	5.5	11.3	6.5	5.2
Carbohydrate^b^	444.1	304.6	469.3	438.8	359.5	210.5	238.3
Gross energy (kJ/g)	19.9	22.3	19.4	19.5	19.2	19.8	21.8
Amino acids profile (g/kg in protein)^c^							
Arg	48.7	32.4	117.7	98.7	49.3	76.9	77.1
His	22.6	20.5	30.9	19.3	43.9	27.3	23.9
Ile	58.7	68.6	32.4	35.4	33.6	44.9	44.9
Leu	97.9	177.3	64.1	58.1	61.3	77.6	79.4
Lys	72.2	17.2	50.8	30.0	36.1	61.4	52.6
Met	16.1	24.0	14.3	6.2	16.3	14.2	12.9
Cys	11.1	5.5	17.6	4.7	23.0	14.4	12.5
Phe	73.3	69.0	57.4	56.4	36.6	50.4	49.6
Tyr	3.55	49.3	27.6	31.5	26.6	32.7	33.3
Thr	50.1	45.1	30.9	25.3	37.9	39.2	40.6
Val	70.2	51.9	49.8	39.7	22.5	46.0	47.9
Ala	39.1	90.5	47.2	34.1	43.9	46.5	42.1
Asp	69.6	70.1	95.2	114.4	70.7	112.2	110.9
Glu	104.3	196.0	200.9	191.2	175.4	171.1	175.9
Gly	46.4	27.7	47.2	51.9	62.9	57.5	42.8
Pro	42.0	130.6	62.5	58.6	47.7	86.1	52.3
Ser	45.3	59.3	46.2	53.0	47.4	43.3	51.1

Abbreviations: BY, beer yeast; CGM, corn gluten meal; CSM, cottonseed meal; PNM, peanut meal; RSM, rapeseed meal; SBM, soybean meal; SPC, soy protein concentrate.

^a^Provided by Shandong Conqueren Marine Technology Co., Ltd., Weifang, China.

^b^Carbohydrate was calculated by difference.

^c^Tryptophan could not be analyzed because of its destruction during acid hydrolysis.

**Table 3:** Proximate and amino acid composition (g/kg) of the reference and test diets.

Items	Reference diet	Test diets (a 70:30 mixture of the reference diet to test ingredient)						
		BY	CGM	CSM	PNM	RSM	SBM	SPC
Proximate analysis (g/kg dry matter)								
Dry matter	897.7	902.5	907.6	913.4	915.0	905.7	912.1	910.2
Crude protein	498.0	500.2	542.9	480.8	501.8	473.2	497.8	560.4
Crude lipid	223.2	157.8	165.9	162.6	163.7	161.1	162.9	161.3
Ash	123.3	99.9	91.0	106.9	106.0	107.3	105.5	98.0
Phosphorus	14.6	13.2	12.5	13.6	11.9	13.6	12.2	11.8
Gross energy (kJ/g)	22.6	21.8	22.5	21.6	21.7	21.6	21.8	22.4
Amino acids profile (g/kg in protein)^a^								
Arg	63.0	58.7	52.1	78.1	73.9	59.4	67.2	68.4
His	22.6	22.6	21.9	24.9	21.6	28.2	24.0	23.1
Ile	43.2	47.9	52.3	40.2	40.8	40.7	43.7	43.8
Leu	67.7	76.9	106.9	66.7	64.8	66.0	70.7	72.1
Lys	74.2	73.6	53.8	67.8	60.7	64.2	70.4	66.1
Met	28.2	24.5	26.7	24.3	21.5	25.0	24.0	22.4
Cys	9.3	9.8	7.9	11.6	7.9	12.9	10.8	10.5
Phe	47.0	54.8	54.7	49.7	49.7	44.1	47.8	47.8
Tyr	38.0	27.4	42.0	35.1	36.0	35.0	36.4	36.2
Thr	45.7	47.1	45.5	41.7	39.5	43.7	43.8	43.8
Val	46.6	53.8	48.5	47.5	44.5	40.3	46.4	47.1
Ala	65.6	57.5	74.5	60.5	55.9	59.9	59.8	56.7
Asp	93.3	87.2	85.0	93.8	99.7	87.3	98.9	99.9
Glu	155.8	140.2	170.2	168.2	166.6	161.0	160.4	163.4
Gly	66.4	60.3	52.5	61.1	62.0	65.4	63.7	57.4
Pro	52.5	49.3	80.4	55.2	54.3	51.2	62.5	52.4
Ser	41.5	42.6	47.8	42.8	45.0	43.0	41.9	45.1

Abbreviations: BY, beer yeast; CGM, corn gluten meal; CSM, cottonseed meal; PNM, peanut meal; RSM, rapeseed meal; SBM, soybean meal; SPC, soy protein concentrate.

^a^Tryptophan could not be analyzed because of its destruction during acid hydrolysis.

**Table 4:** Apparent digestibility (%) of nutrients in the reference and test diets for Coho salmon (*Oncorhynchus kisutch*) postsmolts.

Items	Reference and test diets							
	REF	BY	CGM	CSM	PNM	RSM	SBM	SPC
Dry matter	67.43 ± 1.34^b,c^	68.26 ± 0.35^a,b^	67.37 ± 0.44^b,c^	61.06 ± 0.45^d^	66.92 ± 0.77^b,c^	60.20 ± 0.31^d^	66.21 ± 0.33^c^	69.07 ± 0.48^a^
Crude protein	86.75 ± 1.17^a^	85.64 ± 0.72^a^	84.89 ± 0.56^a^	82.72 ± 0.84^b^	85.38 ± 1.19^a^	82.53 ± 0.64^b^	86.40 ± 0.87^a^	86.75 ± 0.56^a^
Crude lipid	88.01 ± 0.46^a^	87.18 ± 0.75^a,b^	85.99 ± 0.59^b,c^	85.57 ± 0.61^c^	87.04 ± 0.43^a,b^	84.73 ± 0.55^c^	87.56 ± 0.19^a^	87.92 ± 0.36^a^
Phosphorus	72.05 ± 0.71^a^	64.57 ± 0.50^b^	63.98 ± 0.96^b^	61.62 ± 1.14^c,d^	63.41 ± 1.05^b,c^	61.28 ± 1.02^d^	64.64 ± 0.28^b^	63.74 ± 0.68^b^
Gross energy	79.29 ± 0.56^a^	77.05 ± 1.06^b^	74.46 ± 0.84^c,d^	72.79 ± 0.63^c^	76.69 ± 0.98^b^	72.14 ± 0.40^d^	77.50 ± 0.23^a,b^	77.74 ± 0.94^a,b^
Amino acids^1^								
Arg	88.03 ± 0.42^a,b^	88.35 ± 0.56^a^	86.97 ± 0.46^b,c^	86.33 ± 0.72^c,d^	88.64 ± 0.46^a^	85.68 ± 0.39^d^	88.30 ± 0.39^a^	89.11 ± 0.64^a^
His	86.84 ± 1.02^a^	86.31 ± 0.58^a,b^	85.89 ± 0.98^a,b^	84.33 ± 0.36^b,c^	86.52 ± 0.95^a,b^	83.67 ± 1.20^c^	86.29 ± 1.20^a,b^	86.68 ± 0.22^a,b^
Ile	86.75 ± 0.66^a^	86.46 ± 0.72^a^	85.95 ± 0.42^a,b^	84.22 ± 0.87^b,c^	86.08 ± 1.05^a,b^	83.85 ± 0.15^c^	86.89 ± 0.54^a^	86.74 ± 1.16^a^
Leu	85.09 ± 0.48^a^	84.55 ± 0.71^a^	84.59 ± 1.00^a^	82.77 ± 0.91^b^	85.22 ± 0.69^a^	82.26 ± 0.62^b^	85.31 ± 0.61^a^	85.68 ± 0.17^a^
Lys	88.04 ± 0.49^a^	87.41 ± 0.93^a^	86.71 ± 0.93^a^	83.23 ± 0.52^b^	86.27 ± 0.50^a^	82.83 ± 0.81^b^	87.81 ± 0.79^a^	88.06 ± 0.55^a^
Met	85.81 ± 0.60^a^	86.00 ± 1.15^a^	85.61 ± 1.23^a^	82.13 ± 0.73^b^	86.36 ± 0.80^a^	80.97 ± 0.66^b^	85.90 ± 0.92^a^	85.71 ± 0.95^a^
Cys	84.99 ± 0.76^a^	85.06 ± 0.67^a^	85.75 ± 1.00^a^	81.12 ± 0.56^b^	85.15 ± 0.41^a^	81.17 ± 0.14^b^	84.51 ± 0.31^a^	85.15 ± 0.67^a^
Phe	87.65 ± 0.70^a^	87.28 ± 0.23^a^	86.94 ± 1.18^a^	83.27 ± 1.21^b^	87.02 ± 0.31^a^	83.74 ± 0.19^b^	86.80 ± 1.03^a^	87.50 ± 0.78^a^
Tyr	83.60 ± 0.92^a^	82.13 ± 0.55^a,b^	83.20 ± 0.28^a^	81.03 ± 0.54^b,c^	82.77 ± 0.32^a^	80.45 ± 0.66^c^	83.05 ± 0.69^a^	83.18 ± 0.95^a^
Thr	85.17 ± 0.70^a^	84.59 ± 0.61^a^	84.31 ± 0.53^a^	81.65 ± 0.54^b^	84.70 ± 0.23^a^	81.57 ± 0.50^b^	84.47 ± 1.13^a^	84.32 ± 0.70^a^
Val	85.31 ± 0.87^b^	85.44 ± 0.17^b^	85.56 ± 0.36^b^	83.22 ± 0.62^c^	86.26 ± 0.07^a,b^	82.82 ± 0.35^c^	85.90 ± 0.49^a,b^	86.82 ± 0.19^a^
Ala	86.56 ± 0.71^a^	86.71 ± 0.66^a^	86.57 ± 1.03^a^	83.29 ± 0.40^b^	85.84 ± 0.36^a^	83.41 ± 0.46^b^	86.15 ± 0.64^a^	86.00 ± 0.58^a^
Asp	86.33 ± 0.66^a^	86.08 ± 0.95^a^	86.32 ± 0.76^a^	82.74 ± 0.55^b^	86.24 ± 0.24^a^	82.45 ± 0.41^b^	86.02 ± 0.95^a^	86.28 ± 1.03^a^
Glu	82.81 ± 0.49^a^	82.03 ± 0.82^a,b^	82.32 ± 0.41^a^	81.14 ± 0.46^a,b^	82.78 ± 0.69^a^	80.55 ± 0.76^c^	82.05 ± 0.98^a,b^	82.42 ± 0.38^a^
Gly	83.82 ± 0.78^a^	83.47 ± 0.11^a^	83.70 ± 0.95^a^	80.90 ± 1.33^b^	83.37 ± 0.56^a^	80.72 ± 0.68^b^	83.99 ± 0.32^a^	83.63 ± 1.30^a^
Pro	85.81 ± 0.98^a^	85.65 ± 0.77^a^	85.28 ± 0.28^a^	83.08 ± 0.47^b^	86.02 ± 0.52^a^	82.70 ± 0.17^b^	86.08 ± 0.52^a^	86.19 ± 0.41^a^
Ser	84.87 ± 0.77^a^	85.01 ± 0.28^a^	85.45 ± 1.26^a^	82.24 ± 0.15^b^	85.02 ± 0.51^a^	82.32 ± 0.32^b^	84.98 ± 0.25^a^	85.68 ± 1.17^a^

*Note:* Means ± S.D. (*n* = 3) with the same superscript small letters in the same row are not significantly different (*p*  > 0.05), REF, reference diet;.

Abbreviations: BY, beer yeast; CGM, corn gluten meal; CSM, cottonseed meal; PNM, peanut meal; RSM, rapeseed meal; SBM, soybean meal; SPC, soy protein concentrate.

^1^Tryptophan could not be analyzed because of its destruction during acid hydrolysis.

**Table 5:** Apparent digestibility (%) of nutrients in the test ingredients for Coho salmon (*Oncorhynchus kisutch*) postsmolts.

Items	Test ingredients						
	BY	CGM	CSM	PNM	RSM	SBM	SPC
Dry matter	70.19 ± 3.35^a^	67.25 ± 2.93^a^	46.19 ± 2.82^c^	65.73 ± 1.95^a,b^	43.34 ± 3.08^c^	63.38 ± 2.56^b^	72.90 ± 2.05^a^
Crude protein	83.04 ± 2.47^a^	80.56 ± 1.83^a^	73.32 ± 2.05^b^	82.17 ± 2.52^a^	72.69 ± 2.32^b^	85.57 ± 2.16^a^	86.76 ± 2.09^a^
Crude lipid	85.23 ± 1.90^a^	81.27 ± 1.57^a,b^	79.88 ± 2.31^a,b^	84.77 ± 2.02^a^	77.09 ± 1.77^b^	86.51 ± 1.66^a^	87.72 ± 1.87^a^
Phosphorus	47.12 ± 2.57^a^	45.13 ± 2.21^a^	37.29 ± 2.17^b^	43.23 ± 1.85^a^	36.14 ± 2.78^b^	47.35 ± 2.42^a^	44.34 ± 1.67^a^
Gross energy	71.81 ± 2.42^a^	63.19 ± 1.69^b^	57.63 ± 2.31^c^	70.63 ± 2.10^a^	55.44 ± 2.05^c^	73.33 ± 1.95^a^	74.12 ± 1.92^a^
Amino acids^1^							
Arg	89.10 ± 1.78^a^	84.52 ± 1.11^b^	82.38 ± 1.51^b^	90.06 ± 1.22^a^	80.22 ± 1.15^b^	88.94 ± 1.72^a^	91.63 ± 1.86^a^
His	85.08 ± 1.51^a^	83.70 ± 0.93^a^	78.46 ± 1.21^a,b^	85.76 ± 2.12^a^	76.27 ± 1.72^b^	85.01 ± 1.78^a^	86.32 ± 2.11^a^
Ile	85.79 ± 1.98^a^	84.09 ± 2.48^a^	78.31 ± 1.95^b^	84.52 ± 2.33^a^	77.07 ± 1.76^b^	87.23 ± 1.85^a^	86.71 ± 2.33^a^
Leu	83.29 ± 1.64^a^	83.43 ± 2.32^a^	77.36 ± 2.12^b^	85.51 ± 2.10^a^	75.66 ± 2.63^b^	85.83 ± 2.28^a^	87.05 ± 1.53^a^
Lys	85.96 ± 2.16^a^	83.60 ± 1.95^a^	72.01 ± 2.46^b^	82.16 ± 2.76^a^	70.69 ± 1.65^b^	87.28 ± 2.61^a^	88.13 ± 2.84^a^
Met	86.42 ± 2.43^a^	85.14 ± 2.71^a^	73.52 ± 2.13^b^	87.64 ± 2.48^a^	69.66 ± 2.59^b^	86.11 ± 1.70^a^	85.46 ± 1.88^a^
Cys	85.22 ± 2.10^a^	87.53 ± 2.07^a^	72.09 ± 2.63^b^	85.53 ± 2.21^a^	72.26 ± 2.11^b^	83.39 ± 2.75^a^	85.53 ± 2.50^a^
Phe	86.44 ± 2.21^a^	85.28 ± 2.35^a^	73.07 ± 2.41^b^	85.55 ± 1.85^a^	74.64 ± 2.21^b^	84.81 ± 1.92^a^	87.16 ± 2.39^a^
Tyr	78.70 ± 2.76^a,b^	82.26 ± 2.27^a^	75.02 ± 2.19^b^	80.84 ± 1.78^a^	73.11 ± 2.34^b^	81.76 ± 1.97^a^	82.19 ± 2.09^a^
Thr	83.22 ± 2.22^a^	82.28 ± 2.76^a^	73.42 ± 2.75^b^	83.60 ± 1.95^a^	73.16 ± 2.62^b^	82.84 ± 2.17^a^	82.33 ± 2.21^a^
Val	85.75 ± 2.52^a^	86.15 ± 2.21^a^	78.36 ± 1.70^b^	88.47 ± 2.14^a^	77.01 ± 2.78^b^	87.30 ± 1.82^a^	90.34 ± 2.35^a^
Ala	87.07 ± 2.93^a^	86.59 ± 1.94^a^	75.66 ± 2.47^b^	84.16 ± 2.60^a^	76.07 ± 1.63^b^	85.20 ± 2.16^a^	84.68 ± 2.26^a^
Asp	85.52 ± 2.75^a^	86.32 ± 2.39^a^	74.38 ± 2.67^b^	86.03 ± 2.14^a^	73.42 ± 2.65^b^	85.29 ± 2.82^a^	86.17 ± 2.79^a^
Glu	80.21 ± 1.83^a^	81.20 ± 2.38^a^	77.26 ± 2.46^a,b^	82.73 ± 1.72^a^	75.28 ± 2.80^b^	80.30 ± 2.33^a^	81.51 ± 2.35^a^
Gly	82.65 ± 2.17^a^	83.42 ± 2.44^a^	74.09 ± 2.67^b^	82.32 ± 2.39^a^	73.51 ± 2.28^b^	84.38 ± 2.88^a^	83.19 ± 2.50^a^
Pro	85.28 ± 2.39^a^	84.04 ± 1.92^a^	76.72 ± 1.95^b^	86.53 ± 2.09^a^	75.44 ± 1.78^b^	86.72 ± 2.65^a^	87.07 ± 2.85^a^
Ser	85.34 ± 1.87^a^	86.80 ± 2.67^a^	76.11 ± 2.27^b^	85.37 ± 2.62^a^	76.37 ± 2.34^b^	85.24 ± 2.60^a^	87.56 ± 2.14^a^

*Note:* Means ± S.D. (*n* = 3) with the same superscript small letters in the same row are not significantly different (*p*  > 0.05).

Abbreviations: BY, beer yeast; CGM, corn gluten meal; CSM, cottonseed meal; PNM, peanut meal; RSM, rapeseed meal; SBM, soybean meal; SPC, soy protein concentrate.

^1^Tryptophan could not be analyzed because of its destruction during acid hydrolysis.

## Data Availability

All the data in the article are available from the corresponding author upon reasonable request.
